# Processing multisource feedback during residency under the guidance of a non-medical coach

**DOI:** 10.5116/ijme.5a7f.169d

**Published:** 2018-02-23

**Authors:** Caroline A.M. Buis, Marina A.W. Eckenhausen, Olle ten Cate

**Affiliations:** 1 Northwest Clinics, the Netherlands; 2 Center for Research and Development of Education, University Medical Center Utrecht, the Netherlands

**Keywords:** Multisource feedback, residents, coaching

## Abstract

**Objectives:**

The present study aimed to investigate residents’ preferences in dealing with personal multi-source feedback (MSF) reports with or without the support of a coach.

**Methods:**

Residents employed for at least half a year in the study hospital were eligible to participate. All 43 residents opting to discuss their MSF report with a psychologist-coach before discussing results with the program director were included. Semi-structured interviews were conducted following individual coaching sessions. Qualitative and quantitative data were gathered using field notes.

**Results:**

Seventy-four percent (n= 32) preferred sharing the MFS report always with a coach, 21% (n= 9) if either the feedback or the relationship with the program director was less favorable, and 5% (n=2) saw no difference between discussing with a coach or with the program director. In the final stage of training residents more often preferred the coach (82.6%, n=19) than in the first stages (65%, n=13). Reasons for discussing the report with a coach included her neutral and objective position, her expertise, and the open and safe context during the discussion.

**Conclusions:**

Most residents preferred discussing multisource feedback results with a coach before their meeting with a program director, particularly if the results were negative. They appeared to struggle with the dual role of the program director (coaching and judging) and appreciated the expertise of a dedicated coach to navigate this confrontation. We encourage residency programs to consider offering residents neutral coaching when processing multisource feedback.

## Introduction

While multisource feedback (MSF) is considered crucial for the development of competencies during workplace learning in health care, the effect of the feedback depends on the resident’s reception. While the skill to provide feedback has received much attention in the literature receiving feedback may be a skill that is even more difficult to acquire.^1^^-^^5^ Receiving feedback is increasingly viewed as informed self-assessment and may require training with coaching.^6^ Besides, if feedback reports of medical trainees are to be discussed directly with clinical supervisors, they may view MSF as a tool for judgment rather than for development, resulting in less open discussions and thereby limiting reflection and learning. Clinical supervisors, on the other hand, may be hampered in their capacity to be a coach by the fact that they also have to judge a medical trainee’s performance. In general, it has been suggested that the current dual role of clinical supervisors as coach and judge is likely to fail the needs of physicians-in-training by not helping them to reach their full potential.^7^

Feedback in clinical training can be operationally defined as ‘specific information about the comparison between a trainee’s observed performance and a standard, given with the intent to improve the trainee’s performance’.^8^ It is considered crucial for the development of competence during workplace learning.^9^^-^^11^ However, it is not always possible to arrange frequent observation of learners in the clinical workplace. Consequently, clinical teachers may only observe part of the performance that is important for the development of clinical competence. Observation of learner behavior can be enhanced by including feedback from other sources than clinical teachers, such as nursing staff, peers and patients, which is what happens in Multisource feedback (MSF) or 360-degree employee evaluation.

MSF is a questionnaire-based assessment method in which peers, patients, and coworkers evaluate key performance behaviors of trainees. The method is widely used in industrial settings to assess performance and has also gained widespread acceptance as a quality improvement method in health systems.^12^ For example, the College of Medical Specialties in the Netherlands highly recommends multisource feedback for all residency programs. At present, MSF has been introduced in most residency training programs throughout the Netherlands.

In business, which has a longer history of using MSF, there is extensive literature about the best conditions for using this instrument effectively. Carson has recommended connecting MSF processes to organizational goals and strategies, using multisource feedback only for professional development and not for evaluation, training the respondents in giving feedback, using a validated measurement instrument, creating an environment of trust and confidentiality and, last but not least, appointing coaches and mentors to support feedback recipients.^13^

While the technical requirements of MSF instruments have been investigated extensively, little attention has been directed to the reception of multisource feedback in medical education. Participants in a study of Sargeant suggested reviewing their MSF report with an informed professional because that could help them gain insight and identify overlooked areas for improvement. Recommendations for facilitated feedback are consistent with the developmental intent of MSF and with suggestions from industry to appoint ’coaches’ for guiding feedback use.^2^ In line with these suggestions, a pilot study introducing a neutral coach to support residents during the MSF procedure was started in January 2010 by the department of surgery of the Northwest Clinics, the Netherlands. The results of this pilot study inspired six other program directors also to start a multisource feedback procedure with similar support. At present, these residency programs still employ the services of an independent, experienced coach who assists the residents in understanding the feedback and utilizing it for their professional development.

### Description of the MSF tool and procedure

#### Standard Procedure

The MSF tool applied was developed for postgraduate medical education at University Medical Center Utrecht^14^^,^^15^ and consists of a web-based, stepwise procedure. After registering to create an account, the program director (PD) enrolls residents in the MSF program account. Residents then receive an e-mailed request to supply e-mail addresses of multiple observers in 3 categories: medical colleagues (6 or more), other healthcare colleagues (6 or more), and patients (10 or more). These observers, in turn, receive an email request to complete a questionnaire with closed format questions and open boxes for narrative feedback as ‘tops’ (compliments) and ‘tips’ (suggestions for improvement). The questionnaire must be completed and returned prior to a preset closure date, usually a month or longer ahead. At this date, data from all returned questionnaires are aggregated generating a report that is automatically sent to both the resident and the PD, allowing both to prepare for discussion of the findings as noted below. Quantitative data are summarized in a table, categorized according to the CanMEDS framework, and followed by a list of ‘tops’ and ‘tips’.^16^ While the sources of the individual responses remain hidden to the resident, the PD may identify respondents if necessary for a limited period. Next, the full MSF procedure stipulates that the PD and the resident discuss the report during the resident’s progress interview.^14^

#### Adapted Procedure

In the present study, we adapted the standard procedure in five ways. First, the PD asks the coach, a trained psychologist, to initiate MSF procedures for the selected residents in his or her program. The coach and the PD discuss when to start and which residents to include. Second, the coach then informs the residents about the purpose and procedure of the MSF, their role, the independent and confidential role of the coach, and the role of the MSF respondents: to provide narrative, specific, clear and behaviourally tuned feedback. Third, the aggregated report for each resident is sent to the coach, not the PD.  Fourth, prior to the resident receiving the report, the coach checks the data for accuracy, analyses and interprets each report, and paying attention to remarkable or contradictory feedback. Fifth, the coach, not the PD, discusses the report with the resident. In this meeting, the coach starts by asking the resident’s opinion and recognition of the feedback. Examples of probing questions are: ‘What strikes me are your dedication, your empathy, your helpfulness and your perfectionism. How do you keep a balance in doing what is good for you and what is good for others?’ Through discussion, the coach and resident determine learning objectives and a plan how to achieve them. If residents had negative feelings about or did not understand the feedback, the coach offers to facilitate further discussion. If a resident agrees the coach can approach a respondent to ask for clarification of particular feedback while guarding the respondent’s anonymity if he or she wishes so. If the respondent provides consent to reveal his or her identity, the medical residents contacted the relevant respondents. The aim of contacting the respondent was clarification and enhancement of acceptance of feedback. The resulting plan is then discussed with the PD during the medical resident’s progress interview. The content of the discussion with the coach and the MSF report remains confidential. It is up to the resident to share the report with the PD or not. The present study aimed to investigate residents’ preferences in dealing with personal MSF reports with or without the support of a coach.

## Methods

### Study design

The qualitative study was conducted in a large teaching hospital serving 90 medical residents in 25 residency programs. A semi-structured interview technique was used for collecting data. The PDs from seven specialties applied voluntarily to start with the adapted procedure. All participating medical residents and PDs were informed that the procedure would be evaluated. The residents were selected by their PD and needed to have been employed in the hospital for at least half a year. During the period April 2010 until December 2014, the adapted MSF procedure was initiated with 72 individual residents. Following the MSF report discussion between the coach and the resident, the coach invited some of the residents to participate in the study. Residents were purposefully sampled from the seven different participating specialties. When residents agreed, the semi-structured interview was conducted directly after the MSF report discussion. During the interview, the coach took notes. Immediately after each interview, the coach transcribed these field notes into complete sentences, which served as data for the qualitative analysis. After we found saturation of information, we stopped planning new interviews for the study.

### Participants

A total of 43 medical residents were invited to the study and answered the questions shown in Appendix 1. Clinical training programs included were General Surgery, Internal Medicine, Emergency Medicine, Clinical Pharmacy, Pulmonology, Mental Rehabilitation, and Orthopedics.

Ethical approval was sought, and the study was deemed exempted from full review because there was no question of subjecting subjects to actions or conducting behavior as referred to in the definition of medical scientific research in Dutch Act on Experiments with Humans.

We reassured residents that only the data collected in the semi-structured interviews would be used in the report. All participating residents were informed in advance that the adapted procedure would be anonymously evaluated. Interview responses were aggregated anonymously, and direct quotations were de-identified of any information that could be associated with individual participants. Informed consent was given by all study participant to write an article of this study.

### Data collection and analysis

#### Evaluation Instrument Development

The coach created a questionnaire containing questions about MSF instructions, reports received, the MSF instrument, and coaching procedure (see Appendix 1). Two colleagues and one program director commented on the questionnaire which led to minor adaptations. The coach used the revised questionnaire in a 15-minute semi-structured oral interview. All answers were recorded on paper by the coach. Quantitative responses to age, gender, year of residency training and medical specialty were aggregated anonymously and were analysed using SPSS 20 software. For the purpose of this study, we focused the analyses on the questions ‘With whom you the validity of the interpretations, the answers were also categorized by two other persons, the second author and a person not familiar with the context of residents.want to discuss the report?’ and ‘Why?’ Responses to the first question led to answers that could be clustered into categories. It just turns out that the categories for the “with whom” question are discrete and allowed for quantification. To support validity of the interpretations, the answers were also categorized by two other persons, the second author and a person not familiar with the context of residents.

Answers could include more dimensions. Differences were discussed and the notations from the interview were used to clarify the answers. After discussions among the team, it was concluded that the category ‘Neutral and objective’ was related to the neutral position of the coach in the organization. The category ‘Expertise’ was related to the competence of the coach. And the third category ‘Open and safe’ included answers where they refer to the open and safe nature of the conversation and where they refer to the dual role of program directors.

Although not everybody agreed that some answers could include more than one category we agreed on the order; which category is mentioned the most, the second and the least.

## Results

All invited residents (43) participated in the study. The average age of this group was 31.5 years, 39.5% were men, and 60.5% were women, which is comparable to the overall Dutch medical resident population 2010-2014 (men 39.2%, women 60.8%, Capaciteitsorgaan 2016). The distribution of the participants was 34.7% surgical specialties, 61.2% medical specialties and 4.1% supportive specialties (clinical pharmacy, emergency medicine). This distribution is consistent with the purposive sampling.

As the focus of our study was on the role of the coach we focused on question 11: ‘With whom do you prefer to discuss the results of the multisource feedback?’ Seventy-four percent answered that they preferred to discuss the results with the coach 21% said that they could have had the conversation with their PD but if conditions were different they would prefer a coach and 5% answered that they could have had the discussion with their PD.

If residents preferred a coach the answers to the question ‘Why?’ appeared to cluster in three categories: ‘a coach is neutral and objective,’ ‘a coach has specific coaching expertise,’ ‘discussing results with a coach feels open and safe.’

The most frequently mentioned reason why residents preferred a coach was the expertise of the coach (45%) followed by the neutral and objective position of the coach (36%). The least mentioned reason is the open and safe nature of the conversation (19%). Narrative answers in the category ‘expertise’ included: ‘‘I appreciate your open view because that gives me new insights and I appreciate your expertise in guiding medical residents; I don’t have to explain certain situations because you understand them’’(R9). ‘‘With my PD I would not have come to this insight’’ (R40). ‘‘There is more depth during the discussion’’ (R1). ‘‘Interviews well, asks good questions’’ (R11). ‘‘You let me find my solutions, what I prefer above what my PD does; he tells me how I have to do it, and that is not always the way that suits me’’ (R12).

Examples of answers in the next most frequent category ’Objective and neutral’ are: ‘‘Coach is neutral and objective” (R 22). ‘‘More neutral, open and broad thinking’’ (R28).

The least mentioned category ‘Discussing results with a coach feels open and safe’ included answers where they refer to the open and safe nature of the conversation and where the dual role of de PD is mentioned. For example; ‘‘Different interests can play in the PD, the PD isn’t neutral’’ (R4) or ‘‘I can speak openly, some things I don’t discuss with my program director because that information can be used as an appraisal. If the program director discusses the MSF, the conversation should be separated from the progress interview to prevent mixture with judging’’ (R20).

**Table 1:** Reasons to choose for a coach or not

Preference for first discussion of MSF results	Expertise	Neutrality	Safety
Always with program director (N=2)			“I’m at the end of my training’’ (R5) “I already discuss these topics with my PD” (R29)
With coach only in case of negative results or suboptimal relationship with program director (N=9)	“Neutral coach is still better because of specific advice” (R 32)	“As a resident you always have a dependent relationship, which makes you less open” (R30)	“When feedback is negative and results of the MSF are bad” (R 13) “When my relationship with the PD isn’t optimal it’s less safe to discuss the results with the PD”(R 38)
Always with coach (N=32)	“A coach has specific coaching expertise”	“A coach is neutral and objective”	“Discussing results with coach feels open and safe”

Responses from the residents who would shift their preference from PD to coach if conditions were different and of the residents who preferred the PD throughout are shown in  Table 1.

When we reviewed the backgrounds of the medical residents, we saw that participants from 3 out of 7 specialty programs unanimously preferred the neutral coach (n=22) and one medical specialty said they could have had the discussion with their PD, but they could imagine if conditions were different they might prefer a neutral coach (n=6). The reason they gave was that they had a good relationship with their PD, with whom they had already discussed their personal development. Residents’ preferences were related to the stage of training. Those in the final stage of training more often preferred the coach 82.6% (n=19) than those in the first stage of training 65% (n=13).

**Table 2:** Residents’ preferences in the initial discussion of MSF-reports

Who do you prefer to discuss the results with?		Residents	Residents	Total
		First stage training PGY1-3	Last stage training PGY 3-6	
With program director		7	4	11
	Always With PD	1	1	2
	With PD but if MSF results are negative or relationship with my PD isn’t optimal, with coach	6	3	9
With coach		13	19	32
Total		20	23	43

## Discussion

This study aimed to investigate residents’ preferences in dealing with personal MSF reports with or without the support of a coach. We found that a large majority preferred discussing the report with the coach rather than directly with their PD. This percentage was even higher among senior residents. Reasons given were that the coach was neutral and objective, residents valued the coach’s expertise and felt safe to discuss issues openly. Even the medical residents who said that they could have shared the results directly with their program director spontaneously indicated under which conditions a coach would be preferable.

Although we expected that the neutral position of the coach and the safe and open climate would be the primary reasons to prefer a coach, residents added a third reason, namely the expertise of the coach. This is consistent with what the literature says about effective coaching.^17^De Haan and colleagues showed client perceptions of the outcome of coaching to be significantly related to perceptions of the working alliance, client self-efficacy and perceptions of coaching interventions (‘generalized techniques’) of the coach.^18^ Others found evidence for the central importance of the quality of the working relationship (the ‘working alliance’) as seen from both the client’s and the coach’s perspective.^19^ This indicates that what happens ‘in between’ is of central importance. Coaching is a one to one relationship, but in practice, residents are confronted with more relationships including multiple supervisors. The learning climate of the teaching hospitals, in general, affects the nature of feedback. This is consistent with Gregory and Levy’s view emphasizing the role of context in feedback.^20^

Our results appear to support Cavalcanti and Detsky’s findings that the current dual role of clinical supervisors – coach and judge – is likely to fail the needs of physicians-in-training by not helping them to reach their full potential.^7^

Two reasons to prefer a coach (‘a coach is neutral, and objective’ referring to the neutral position in the organization and the answers in the category ‘discussing results with a coach feels open and safe’) show that residents struggle with this duality. How can a safe environment be created where it is possible for the medical resident to show emotions and to be open in the things he or she wants to improve? Is it possible to separate assessment and development? Literature gives suggestions for a positive feedback environment. Gregory and Levy found that the mere frequency with which a supervisor and subordinate interact predicts the subordinate’s perceptions of the feedback environment: the more they interact, the more positive the feedback environment is experienced, based on subordinates ratings.^21^  These authors show in another study that it is not the moment itself but the whole context which determines the results of feedback.^20^    Many authors have highlighted the importance of a safe and supportive climate for the exchange of feedback. But the specific constituents of a safe climate remain poorly understood, as are the ways in which individual and organizations can promote it.^25^ However, the notion of safe and accepting learning environment does not just refer to the objective characteristics of an environment, but something that is individually experienced; (the expectation) that one feels oneself accepted and that one’s contributions to the discussion will be received. We cannot assume the environment to be or feel the same for everyone.^26^

After discussing the influences of the dual role of the program director and the learning environment, our results also show that residents would prefer a coach if results are negative. We expected to find that medical residents’ preferences to fall into two categories, either ‘Preference for discussing MSF with PD’ or ‘Preference for discussing MSF with a coach’. But a third category emerged: preferring discussions with a coach if either the feedback or their relationship with the program director would be less favorable. This group initially said they could discuss the results with their PD but all participants added spontaneously they would prefer a coach if results are bad or if their relationship with the PD was less favorable. This supports the findings of Kluger and DeNisi, Brett and Atwater and Smither that receiving negative feedback can evoke strong emotional reactions such as anger, shame or powerlessness^22^^-^^24^ which is consistent with the findings of a supportive climate for the exchange of feedback as described earlier.

The preference for a coach seems to be stronger in the second part of residency training (Table 2). We initially expected to find the reverse, as junior residents might feel more insecure and would feel safer discussing the results with someone not related to their training. Further investigation is needed to explore further these preferences of residents in the second part of their training.

The most important question that was raised by the present study is whether program directors should discuss a multisource feedback with their residents at all. Residency program directors are increasingly trained to discuss multisource feedback reports with residents. However, discussing received feedback with a person who also judges you, even if that person is trained in discussing the results properly, may lead to a vulnerable position of the resident.

We think it is important to separate judgment from formative feedback aimed at development. Others have described that learning from feedback is a complex process.^1-6  Considering^ our results, we recommend a coach facilitate discussions of multisource feedback reports with residents. Our advice is in line with Carsons recommendations for using an MSF instrument effectively.^13^

One limitation of our study was that the interviews and their analyses were conducted by the coach (CB). The coach is aware of the fact that she is the main investigator, which can influence the results to the benefit of the coach. It would have been more objective to let another person evaluate the total procedure. To overcome this, the coach asked open-ended questions and emphasized at the beginning of each evaluation that this is not about herself but about having a discussing with a person not related to the discipline and with no role in assessing. We chose to have the coach conduct the interviews immediately after the MSF discussion out of convenience to the residents, sacrificing some objectivity to increase the response rate. The coach/interviewer had no supervisory or evaluative relationship with any of the student participants.

Confirmation bias could have played a part, as the primary investigator (coach and interviewer) could have tended to search for, interpret, focus on and remember information in a way that confirms her preconceptions.^27^ Obtaining permission to record and transcribe verbatim the interviews could have minimized this influence.

Future research should include a larger number of medical residents from different hospitals and should be conducted by an independent investigator. The investigation is still worthwhile because it gives the opinions of residents on a multisource feedback procedure that is broadly used.

## Conclusions

Learning from multisource feedback is a complex process. Most medical residents in our study prefer discussing their multisource feedback report with the coach rather than their program director. Reasons they gave were the neutral and objective position of the coach, the open and safe climate, and the expertise of the coach. This study shows the value of a coach in MSF procedures. We encourage other residency programs to assist residents with multisource processing feedback under the guidance of a non-medical coach.

### Acknowledgements

The authors would like to thank the 43 medical residents who have participated in this study. Thanks are also for Tjeerd van der Ploeg for his help with the data analysis, Lisette van Hulst for text recommendations, Marjan Bakker for her help with the references, Margit Bouma for her help with categorizing the answers, and Dr Judith Bowen from the Oregon Health Sciences University, USA for her assistance in reviewing the language and her invaluable comments on the manuscript.

### Conflict of Interest

The authors declare that they have no conflict of interest.

## Supplementary material

Supplementary file 1 Appendix 1. Survey questions
