# Supplementary material for: Processing multisource feedback during residency under the guidance of a non-medical coach
Source: Int J Med Educ. 2018 Feb 23;9:48–54. doi: 10.5116/ijme.5a7f.169d (PMC5834823; doi:10.5116/ijme.5a7f.169d)
Supplement: Supplementary file 1 — Appendix 1. Survey questions [file ijme-9-48-S1.pdf]

Appendix 1

Survey questions

Information before starting the procedure

1. Was the aim of the MSF clear before you started?
2. Was it clear to you what you had to do?
3. Was the information provided by the program director and the coach clear?
4. Did the information of the program director and the coach match?
5. What did you miss prior to the start of the procedure?

Using MSF

6. Working with the tool MSF, how did that go?
7. Do you have suggestions for improvement?

MSF summary report

8. What did you learn from the results?

Discussing MSF results

9. What did you learn from discussing the results?
10. Was the facilitated discussion an addition to the results?
11. With whom do you prefer to discuss the results? And why?
12. Do you have other suggestions for improvement?
